# Post-treatment Stability in Orthodontic Retention with Twistflex Retainers—Do Patients Benefit from Additional Removable Retainers?

**DOI:** 10.1007/s00784-022-04490-1

**Published:** 2022-04-26

**Authors:** Isabel Knaup, Ulrike Schulte, Jenny Rosa Bartz, Christian Niederau, Rogerio Bastos Craveiro, Andreas Jäger, Michael Wolf

**Affiliations:** 1 Department of Orthodontics, RWTH Aachen University Hospital, Aachen, Germany; 2 Private Dental Office, Cologne, Germany; 3 Department of Orthodontics, Bonn University Hospital, Bonn, Germany

**Keywords:** Twistflex retainers, x-effect, Retention, Hawley retainers, Fixed retainers, Removable retainers, Orthodontic treatment

## Abstract

**Objectives:**

To evaluate post-treatment movements of lower anterior teeth during orthodontic retention in patients with fixed twistflex retainers versus those with combined fixed and removable retainers.

**Materials and Methods:**

This study was based on a retrospective data analysis of 57 adult patients during orthodontic retention. They were assigned to two groups: In group 1 (*n* = 30) the lower jaw was provided with twistflex retainers only and in group 2 (*n* = 27) with a twistflex combined with a removable retainer for night-time use. Orthodontic study models of the lower jaw were digitalized and superimposed. Tooth movements were analyzed at the retainer bonding (t0) and follow-up appointment ≥ six months later (t1). Rotational tooth movements (°) were measured around the x-axis (mesial/distal direction), the y-axis (buccal/lingual direction) and the z-axis (longitudinal direction, tooth axis). Translational tooth movements (mm) were registered along the x-axis (buccal/lingual direction), the y-axis (mesial/distal direction) and the z-axis (apical/coronal direction).

**Results:**

Canine and incisor position changes during orthodontic retention were more pronounced in group 1 compared to group 2 except for canine rotations around the z-axis. In both groups in most of the cases stable lower incisor alignment could be found, but the proportion was significant higher in group 2 (group 1: 56.7% vs. group 2: 81.5%). Severe misalignment was present in 13.3% of the participants of group 1 and only in 7.4% of group 2. The extent of canine tipping and movements along the x- and y-axis in severe misalignment cases was significantly lower in group 2 compared to 1.

**Conclusions:**

Lower incisor alignment was more stable in patients with combined fixed and removable retainers compared to fixed retainers only.

**Clinical Relevance:**

Based on the present findings, the routinely application of supplementary removable retainers can be recommended to enhance anterior tooth alignment in patients with fixed twistflex retainers.

## Introduction

Stabilizing treatment results remains one of the main goals in orthodontic practice. Fixed or removable retention appliances can reduce the risk of relapse [1], but to date there is still limited evidence on treatment protocols, wearing time and duration [2]. However, long-term retention with fixed lingual retainers is commonly recommended and often considered the gold standard (method of first choice) in orthodontic retention [3–7].

**Fig. 1:**
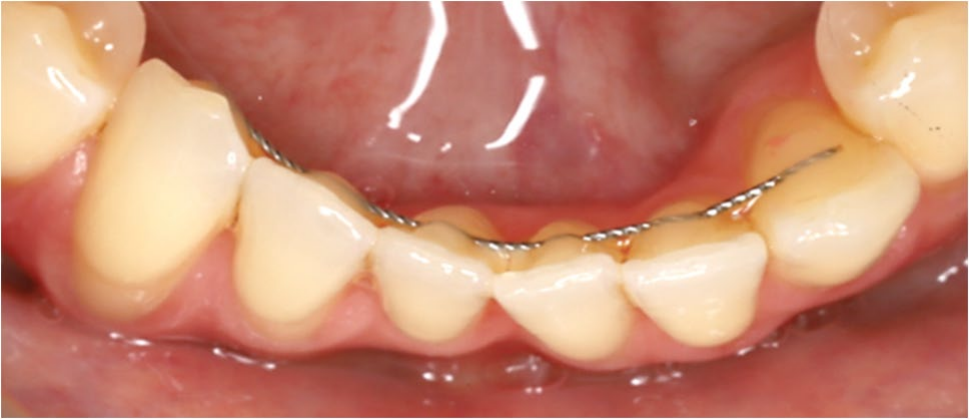
Representative example of retainer-induced tooth misalignment. Intraoral view of a 33-year-old female patient, who presented with a severe reclination of the lower right canine and a proclination of the lower left canine in the presence of a twistflex retainer

Regardless of the expected benefits concerning tooth stabilization [8], aesthetic appearance and independency from the patient’s compliance [9], there have been increasing numbers of reports on undesirable changes in tooth position in the presence of retainers (Fig. 1) [10–15]. Since “*active”* lingual retainers are able to exert forces to teeth [16–18], they have been suspected of causing torque changes of adjacent incisors (“X-effect”) or opposite inclinations of contralateral canines (“twist effect”) [12, 14, 15]. Klaus et al. have found unwanted tooth movements to occur more frequently in cases with retainers in the maxilla than in those in the mandible, in patients with oral dysfunctions/habits and in teeth without interincisal contacts [19]. Retainer associated misalignment has been identified as its own entity, which has to be considered separately from normal developmental changes or relapse during orthodontic retention [10, 20] and, in many cases, leads to a subsequent orthodontic treatment need [21].

As fixed retainers show a higher risk of failure in the upper than in the lower jaw, most clinicians insert maxillary removable and mandibular fixed retainers as their standard retention procedure [22]. In general, Hawley and vacuum-formed retainers are the most common removable retainers in orthodontic retention. Hawley retainers show some advantages over vacuum-formed retainer such as better vertical tooth settling [23], but they are more frequently associated with incisor irregularity and discomfort [24–26]. However, Vagdouti et al. found good compliance for both appliances over a short-term retention phase [27]. Another advantage of removable retainers is their ability to maintain the arch widths. Therefore, several authors recommend to insert removable and fixed in both arches for maximum relapse prevention [28].

In that context, the aim of this retrospective study was to evaluate misalignment of lower anterior tooth during orthodontic retention in patients with fixed twistflex retainers versus patients with combined fixed and removable retainers. The study hypothesis was that lower anterior tooth alignment was more stable in cases of fixed and removable retainers in orthodontic retention.

## Materials and methods

### Study design

The study was based on a retrospective data analysis of 57 adult patients (female: 34, male: 23; age: 18–67 years) during orthodontic retention.

### Retrospective controlled clinical study—participants

All patients attending the Department of Orthodontics at University Bonn, Germany, for routine orthodontic examinations between 2012 and 2015 were assessed for eligibility. The study was designed as a retrospective investigation on routinely collected patient data to determine tooth position changes during orthodontic retention with fixed flexible retainers. Since this was a retrospective study with no prior similar investigation, no sample size calculation was performed beforehand. The inclusion criteria were: completed fixed orthodontic treatment (nonextraction and extraction cases), current 6-point fixed lingual retainer (Dentaflex 0.45 mm, round, three-strand twisted steel wire, Dentaurum, Ispringen, Germany) in the lower jaw (canine to canine). Exclusion criteria were extracted or congenitally missing anterior teeth, broken or damaged retainers, retainers with broken bonding pads and patients which reported retainer failures.

In total, 57 patients met the inclusion criteria. They were assigned to two groups: Group 1 (*n* = 30; female: 17, male: 13) was treated with twistflex retainers only, and group 2 (*n* = 27; female: 17, male: 10) was treated with twistflex retainers in combination with removable retainers. No incentives were offered.

### Retention protocols

Retainer insertion was performed on the day of bracket debonding by a clinician specialized in orthodontics. Therefore, the teeth 33–43 were cleaned, 35% phosphoric acid (Vococid®, VOCO® GmbH, Cuxhaven, Germany) was applied for 20 s, the etching agent was removed using water spray, the enamel surface was dried and coated with a primer (Transbond™ XT, 3 M™ Unitek GmbH, Neuss, Germany). Then, a 6-point retainer was inserted using a silicone positioner and a flowable resin (Kanisit Composite, Kaniedenta, Herford, Germany). Surpluses were removed and light curing was performed using a light-emitting diode (LED) device. All retainers were fabricated in the dental laboratory of the Department of Orthodontics at University Bonn, Germany, beforehand by one calibrated dental technician.

**Fig. 2:**
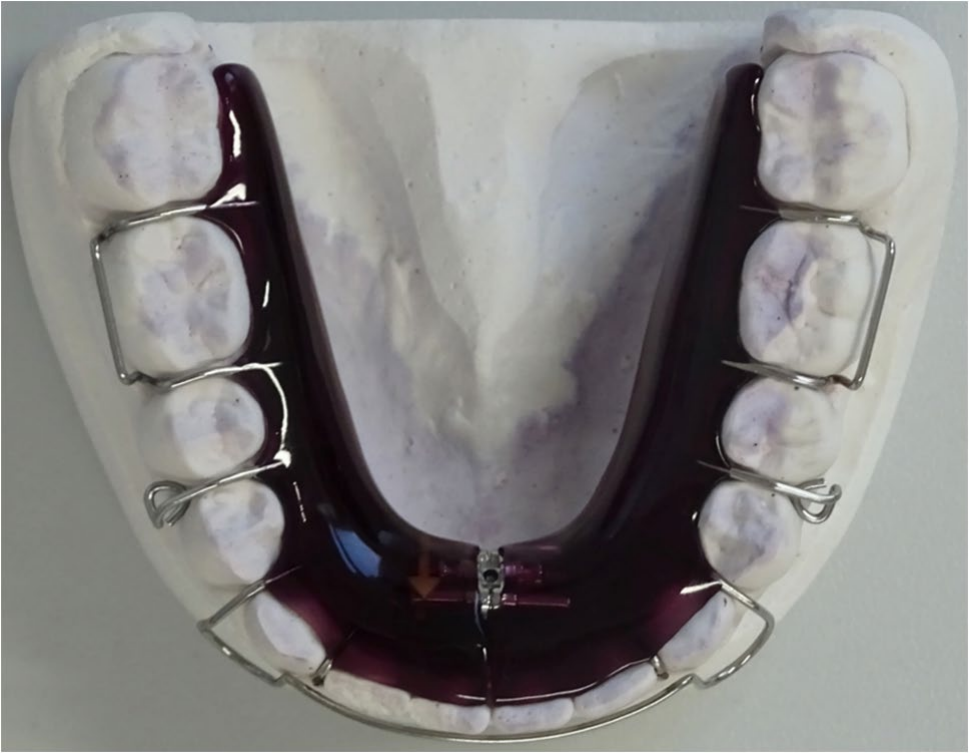
Exemplary illustration of a removable retainer. Participants of study group 2 were provided with twistflex and additional removable retainers for night-time use (incorporated screw was not activated)

Participants of group 2 received an additional removable retainer for night-time use (Fig. 2). The removable retainer consisted of a labial arch, clamps, a resin base and an optional screw and was fabricated in the same dental laboratory. The routinely use of removable retainers depended on the clinician’s decision and was part of his or her standard operating procedure. Incorporated screws were not activated during observational period.

### Clinical examination

Routine consultations of the patients were performed each 6–8 weeks. On the day of retainer insertion (t0) and after ≥ 6 months and ≤ 12 months of retention (t1) the following parameters were recorded as part of the standard treatment protocol:

- Orthodontic study models (stone plaster, BonDur, Wiegelmann, Bonn, Germany) and intraoral photographs were made at the retainer bonding (t0) and follow-up appointment (t1) [14].
- The dental casts were digitalized with a laser scanner (Micromeasure 70®; Microdenta Sensorik, Linden, Germany), and the obtained STL files were superimposed in a 3D graphics software (Surfacer, version 10.5; Imageware/Siemens PLM Software, Plano, USA) to detect positional changes of the teeth. The precision of the laser scanner was shown before [29].

### Superimpositions of the virtual 3D casts

Superimpositions were performed with the software Surfacer (version 10.5; Imageware/Siemens PLM Software, Plano, USA). First, gingiva, retainers and bonding pads on the casts had to be digitally removed as the gingiva might be subjected to dimensional changes and the superimposed teeth had to be displayed in toto for superimposition [14]. Afterward, the virtual casts of t1 and t0 were superimposed using a “best fit method” which was based on an iterative closest point (ICP) matching algorithm. In this algorithm, each point of the 3D point cloud of the digitized model is matched several times with the closest corresponding point of the 3D point cloud of the segmented model. The aim was to achieve an ideal congruence between the premolars and molars of the two models as their position was assumed to be almost stable [14, 30].

**Fig. 3:**
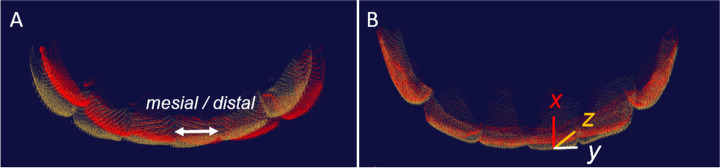
Digital superimpositions of lower anterior teeth. Participants with twistflex retainers (**A**) were compared to participants with twistflex and additional removable retainers (**B**) regarding their misalignment during orthodontic retention. Yellow areas show the teeth at retainer insertion (t0), red areas show them ≥ six months later (t1); tooth position changes were described for the x-, y- and z-axis

To measure possible tooth position changes during orthodontic retention, the teeth 33 to 43 of both study models (t1 and t0) were segmented and possible differences related to rotation and translation were calculated in all three dimensions (Fig. 3) [14, 30].

The following measurements were recorded:

- The coordinate system was defined such that the rotational components of tooth movement (°) in the mesial/distal direction were registered around the x-axis, in the buccal/lingual direction around the y-axis and in the longitudinal direction around the z-axis (= tooth axis, Fig. 3).
- The translational components (mm) were registered in the buccal/lingual direction along the x-axis, in the mesial/distal direction along the y-axis and in the apical/coronal direction (intrusion/extrusion) along the z-axis (Fig. 3).
- Based on the measured tooth movements, all participants were categorized into one of three groups due to the visual appearance of the misalignment: stable anterior alignment (position changes of < 5°), moderate misalignment (position changes of ≥ 5—≤ 9° of at least one tooth) or severe misalignment (position changes > 9° of at least one tooth) [14].

Patients were examined in the Department of Orthodontics by experienced clinicians. All superimpositions were performed by one trained and calibrated examiner. The calibration procedure was performed with 10 superimpositions prior to the beginning of the study. Afterward, a dentist analyzed the virtual models twice on different days to ensure the reproducibility of the data. The average measuring difference was 1° and 0.1 mm [14].

### Statistical analysis

Data were recorded in Microsoft Excel files (Microsoft Office 365, Microsoft Corporation, Redmond, Washington, USA) and transferred to GraphPad Prism (version 7, GraphPad Software, San Diego, California, USA) for analysis and graphic creating.

Continuous data are shown as mean (absolute values) ± standard error (SEM), categorical outcomes as relative frequencies (%). Normality was tested using a Shapiro–Wilk test. The outcome extent of tooth movement was determined by superimposition for tooth tipping (degrees) and tooth movement (millimeters) and compared between the different groups using a two-way ANOVA or a Mann–Whitney U test. The outcome severity index was compared between the different groups using a Chi-squared test. A p-value ≤ 0.05 was predefined to indicate statistically significant differences.

## Results

### Clinical examination

A total of 57 participants with 342 lower teeth (114 canines, 114 second incisors, 114 first incisors) were analyzed as baseline at the time of retainer insertion (t0) and again ≥ six months later (t1) during orthodontic retention. Group 1 (*n* = 30) was exclusively provided with twistflex retainers and group 2 (*n* = 27) with twistflex retainers combined with removable retainers.

### Digital analysis of tipping movements

**Fig. 4:**
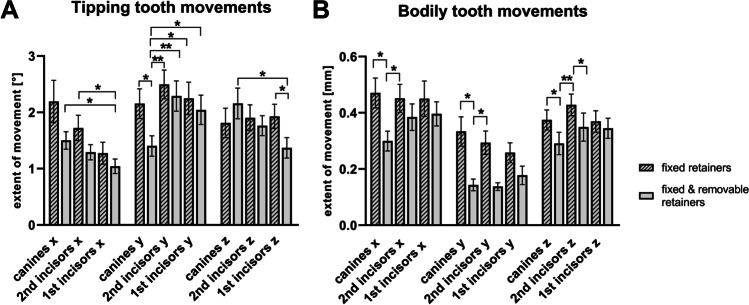
Quantification of misalignment during orthodontic retention with fixed retainers. Results of the superimpositions of lower canines and incisors at retainer insertion and ≥ six months later in participants provided with twistflex (*n* = 30) or twistflex and removable retainers (*n* = 27). Differences are described for the x-, y- and z-axis in degrees (tipping movements, **A**) or millimeters (bodily movements, **B**); mean ± SEM; statistically significant differences are marked with *p ≤ 0.05, **p ≤ 0.01 (Mann–Whitney U test)

Rotations in the mesial/distal direction (x-axis) were more pronounced in group 1 compared to group 2 with decreasing number of frequencies from canines to incisors (canines 2.19 ± 2,91° vs. 1.50 ± 1.14°; second incisors 1.72 ± 1.74° vs. 1.29 ± 1°; first incisors 1.27 ± 1.51° vs. 1.04 ± 0.95°). However, the measured differences were statistically significant only for second incisors of group 1 compared to first incisors of group 2 (Fig. 4A).

Rotations in the buccal/lingual direction (y-axis) were more pronounced in group 1 compared to group 2 (canines 2.16 ± 2.04° vs. 1.40 ± 1.33°; second incisors 2.50 ± 1.98° vs. 2.29 ± 1.97°; first incisors 2.25 ± 2.23° vs. 2.04 ± 1.91°) with significant differences between canines.

First and second incisor rotations around the z-axis were increased in group 2 with significant differences between first incisors of both groups (group 1 vs. group 2: canine 1.81 ± 2.02° vs. 2.16 ± 2.00°; second incisors 1.90 ± 1.78° vs. 1.76 ± 1.30°; first incisors 1.93 ± 1.66° vs. 1.37 ± 1.66°).

### Digital analysis of bodily movements

Bodily canine and incisor movements during orthodontic retention were more pronounced in group 1 compared to group 2 (Fig. 4B).

However, the differences regarding mean buccal/labial movements (x-axis) were significant only in canines (canines 0.47 ± 0.41 mm vs. 0.30 ± 0.26 mm; second incisors 0.45 ± 0.39 mm vs. 0.38 ± 0.35 mm; first incisors 0.45 ± 0.49 mm vs. 0.40 ± 0.32 mm).

Likewise, the extent of mesial/distal movements (y-axis) was significantly higher in canines of group 1 compared to group 2 (canines 0.33 ± 0.40 mm vs. 0.14 ± 0.15 mm; second incisors 0.29 ± 0.32 mm vs. 0.14 ± 0.10 mm; first incisors 0.26 ± 0.27 mm vs. 0.18 ± 0.24 mm).

Moreover, there were significant differences in the vertical direction (z-axis) between the groups regarding canines and second incisors (canines 0.37 ± 0.28 mm vs. 0.29 ± 0.29 mm; second incisors 0.43 ± 0.30 mm vs. 0.35 ± 0.37 mm; first incisors 0.37 ± 0.29 mm vs. 0.34 ± 0.26 mm).

### Severity of misalignment during orthodontic retention

**Fig. 5:**
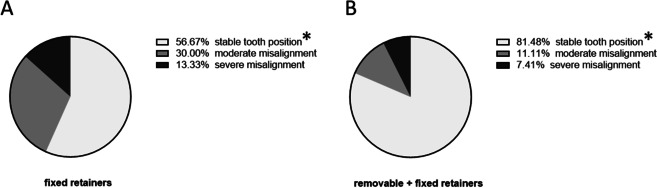
Severity of misalignment during orthodontic retention. Pie charts illustrating the proportions of stable anterior tooth alignment (deviation < 5°), moderate (≥ 5—≤ 9°) or severe (> 9°) misalignment in patients with twistflex (*n* = 30, **A**) or twistflex and removable retainers (*n* = 27, **B**) ≥ six months after retainer insertion. Statistically significant differences are marked with *p ≤ 0.05 (Chi-squared test)

Participants of group 1 (fixed retainers) and group 2 (fixed combined with removable retainers) were assigned to different grades regarding a defined severity index of incisor misalignment (Fig. 5).

Most participants of group 1 (twistflex retainers only) as well as of group 2 (twistflex combined with removable retainers) showed stable lower incisor alignment, but the proportion of stable results was significantly lower in group 1 (56.7 vs. 81.5%). Moderate misalignment occurred in 30% of participants of group 1 and 11.1% of group 2, whereas severe misalignment was present in 13.3% of group 1 and only 7.4% of group 2. Therefore, tooth position was more stable in participants who were provided with twistflex combined with removable retainers during the observational period.

**Fig. 6:**
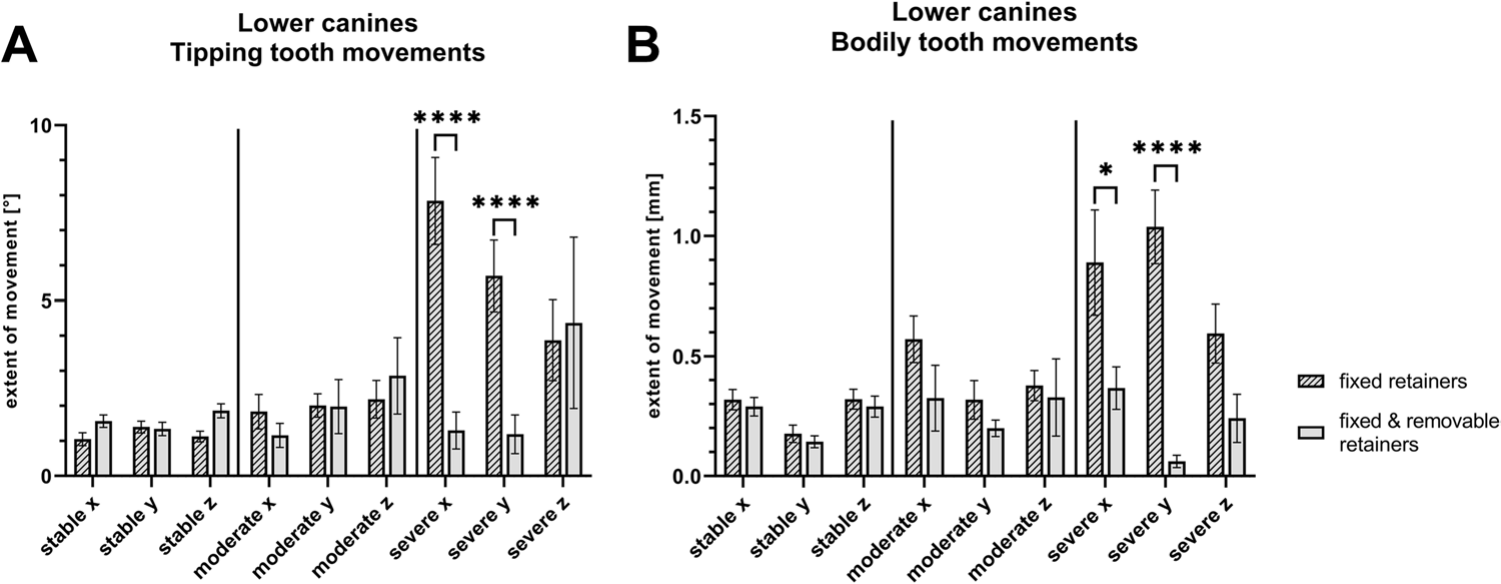
Quantification of canine misalignment during orthodontic retention. Results of the superimpositions of lower canines at retainer insertion and ≥ six months later in participants provided with twistflex (*n* = 30) or twistflex and removable retainers (*n* = 27). Participants were categorized into three groups: stable anterior tooth alignment (deviations < 5°), moderate (≥ 5—≤ 9°) or severe (> 9°) misalignment. Differences are described for the x-, y- and z-axis in degrees (tipping movements, A) or millimeters (bodily movements, B); mean ± SEM; statistically significant differences are marked with *p ≤ 0.05, ****p ≤ 0.0001 (two-way ANOVA test)

Further examination revealed that canine rotations and movements along the x- and y-axes in the severe misalignment cases were significantly lower in group 2 compared to 1 (Fig. 6). Therefore, these patients seem to particularly benefit from the additional insertion of a removable retainer.

## Discussion

The present retrospective investigation evaluated the impact of fixed twistflex retainers on unwanted misalignment of lower anterior teeth during orthodontic retention and it questioned whether the additional insertion of removable retainers reduced the severity of misalignment after at least six months.

Fixed lingual retainers are one of the most common used orthodontic appliances to maintain anterior tooth alignment [31]. Passively inserted, they are claimed to reliably prevent misalignment. However, our results demonstrate that a certain degree of incisor and canine movement even occurred in patients with fixed 6-point retainers and in patients wearing both fixed and removable retainers. This might be explained by the fact that approximately 2.7—5% of patients with retainers made from multistranded wires are affected by unexpected tooth movements during the orthodontic retention phase [10, 32]. Our observations underline the idea that twistflex retainers are able to apply active forces on teeth which could be responsible for iatrogenic tooth movements [15, 16]. Therefore, the observed tooth movements could be more likely part of unwanted tooth movements than part of relapse.

Krämer et al. found that post-orthodontic misalignment mainly occurred during the first 6 months of retention [1]. Hence, the timing of the present follow-up evaluation seems to be adequate.

All patients of the present investigation were provided with twistflex retainers. Some authors assume that these highly flexible twisted retainers show an increased risk to produce inadvertent tooth movement [33]. Engeler et al. demonstrated that plain and braided retainers were more predictable in torsional load transfer than multistranded retainers, which may have stored more energy in the segment between the bonding pads and, therefore, are suspected to induce higher incidences of unexpected tooth movement in clinical use [34]. Moreover, the impact of chewing forces to activate an initially passive retainer is discussed [5, 18].

In the literature, unwanted tooth movements were observed in both, patients with directly and those with indirectly bonded retainers, but the amount of misalignment was lower in patients with indirectly bonded retainers [35]. In the present investigation all retainers were bonded indirectly, so it can be estimated that the extent of unwanted tooth movements could be even higher in patients with directly bonded retainers.

Further reasons for misalignment during orthodontic retention might be found in the orthodontic treatment itself: Intercanine expansion, lower incisor protrusion and mandibular anterior protrusion are claimed to pose a risk for potential misalignment during fixed orthodontic retention [14, 36, 37]. There is a general demand to combine removable and fixed retention appliances in patients with intended dental arch expansion [14, 28]. Our results underline this claim, as we were able to demonstrate fewer misalignment in patients with a combination of fixed and removable retainers compared to patients wearing fixed retainers alone particularly regarding tooth movements along the x- and y-axis.

## Limitations

There are some limitations of this retrospective study to note. Outcomes are based on patients wearing fixed lingual retainers for at least six months and must be regarded as short-term retention findings. Since the study was designed as a retrospective controlled clinical study, no sample size calculation was performed beforehand and the sample size number was low. As prior treatment charts could not be analyzed, it was not possible to specify on which degree the demonstrated tooth movements were part of a relapse to the original position or whether they were retainer associated. Also, there was no study group wearing removable retainers only. It could not be evaluated if these patients showed fewer post-orthodontic misalignment.

It cannot be excluded that during retainer bonding unintended forces were introduced and could have impacted the measured results, even though the retainers were bonded indirectly. Moreover, it was not possible to evaluate the patients’ compliance in wearing the removable retainers.

Therefore, future randomized clinical trials are necessary to understand the exact mechanisms of unwanted tooth movements during orthodontic retention and the findings of this study should be interpreted with caution.

Taken together, the present data show that undesired tooth movement can be expected during orthodontic retention with twistflex retainers. The additional insertion of removable retainers for night-time wear was associated with decreased misalignment.

## Summary

The present retrospective investigation evaluated the impact of removable retainers as a supplement to fixed twistflex retainers on unwanted tooth movement of lower incisors in the orthodontic retention phase. Based on the results it can be summarized:

- Severity of misalignment was lower in participants wearing fixed combined with removable retainers, and canines could be maintained in their position more reliable in severe misalignment cases.
- Based on our findings, the routinely night-time use of removable retainers can be recommended for clinical practice to enhance lower tooth alignment stability in patients with twistflex retainers.
